# Exposure to Environmental Ozone Alters Semen Quality

**DOI:** 10.1289/ehp.8232

**Published:** 2005-10-05

**Authors:** Rebecca Z. Sokol, Peter Kraft, Ian M. Fowler, Rizvan Mamet, Elizabeth Kim, Kiros T. Berhane

**Affiliations:** 1 Department of Obstetrics, Gynecology and Medicine, and; 2 Department of Preventive Medicine, Keck School of Medicine of the University of Southern California, Los Angeles, California, USA; 3 California Cryobank, Los Angeles, California, USA

**Keywords:** air pollution, epididymis, male infertility, ozone, semen analysis, sperm concentration, total motile sperm count

## Abstract

Idiopathic male infertility may be due to exposure to environmental toxicants that alter spermatogenesis or sperm function. We studied the relationship between air pollutant levels and semen quality over a 2-year period in Los Angeles, California, by analyzing repeated semen samples collected by sperm donors. Semen analysis data derived from 5,134 semen samples from a sperm donor bank were correlated with air pollutant levels (ozone, nitrogen dioxide, carbon monoxide, and particulate matter < 10 μm in aerodynamic diameter) measured 0–9, 10–14, and 70–90 days before semen collection dates in Los Angeles between January 1996 and December 1998. A linear mixed-effects model was used to model average sperm concentration and total motile sperm count for the donation from each subject. Changes were analyzed in relationship to biologically relevant time points during spermatogenesis, 0–9, 10–14, and 70–90 days before the day of semen collection. We estimated temperature and seasonality effects after adjusting for a base model, which included donor’s date of birth and age at donation. Forty-eight donors from Los Angeles were included as subjects. Donors were included if they collected repeated semen samples over a 12-month period between January 1996 and December 1998. There was a significant negative correlation between ozone levels at 0–9, 10–14, and 70–90 days before donation and average sperm concentration, which was maintained after correction for donor’s birth date, age at donation, temperature, and seasonality (*p* < 0.01). No other pollutant measures were significantly associated with sperm quality outcomes. Exposure to ambient ozone levels adversely affects semen quality.

Approximately 2.1 million married couples in the United States have reported difficulty in achieving a pregnancy (Abma et al. 1997; Ventura et al. 1999). Male factor infertility accounts for 40–50% of these cases, for which the etiology is often idiopathic (Baker et al. 1995; MacLeod 1951; Sokol 1997; Ventura et al. 1999). Idiopathic male infertility may be due to exposure(s) to environmental toxicants that alter the reproductive hormones, spermatogenesis, or sperm function (Carlsen et al. 1992; Moline et al. 2000; Safe 2000; Sharpe and Skakkebaek 1993).

The most widely studied evidence of potential environmental reproductive hazards is the report that sperm counts have declined in certain industrialized countries (Carlsen et al. 1992; Moline et al. 2000; Safe 2000; Sharpe and Skakkebaek 1993). The validity of these findings continues to be controversial, yet most investigators agree that if a decline in semen quality does exist, these changes are probably related to geographic location (Acacio et al. 2000; Becker and Berhane 1997; Bonde et al. 1998; Bujan et al. 1996; Fisch et al. 1996; Handelsman 1997; Olsen et al. 1995; Paulsen et al. 1996; Rasmussen et al. 1997; Sherins 1995; Swan et al. 1997, 2000). Theories explaining this geographic phenomenon include environmental (chemical exposures, elevated ambient temperature, seasonality), demographic (socioeconomic, ethnicity, age), and methodologic factors (Handelsman 1997; Moline et al. 2000).

Various chemicals have been implicated as reproductive toxicants. A number of these chemicals categorized as air pollutants are present in the blood, urine, and semen of exposed men and may affect sperm quality [Centers for Disease Control and Prevention (CDC) 2001; Fredricsson et al. 1993; Samet et al. 2000; Selevan et al. 2000; Sram et al. 1999; Telisman et al. 2001]. Preliminary data have been published to suggest that air pollution may adversely affect semen quality (Selevan et al. 2000).

The present study was designed to address the hypothesis that exposure to fluctuating levels of specific air pollutants adversely affects sperm parameters. For this purpose, we analyzed repeated semen samples collected by sperm donors in Los Angeles, California, for one sperm bank in relationship to temporally related exposure to air pollutants. Air pollutants of interest were ozone, nitrogen dioxide, carbon monoxide, and particulate matter < 10 μg/m^3^ in aerodynamic diameter (PM_10_). Each of these has a biologic plausibility of affecting sperm production, and the daily levels of these pollutants are measured and recorded for well-demarcated areas throughout Los Angeles County (Gauderman et al. 2000).

We studied the potential impacts of exposure to air pollution during biologically relevant time points during spermatogenesis. Spermatogenesis, which takes place in the seminiferous tubules of the testes, is the orderly process during which spermatogonia evolve into mature spermatozoa. This process covers a 72-day period in humans. The mature spermatozoa then exit the seminiferous tubules, enter the rete testis and efferent ductules, and move into the epididymis, where they are rendered motile and fertile and are stored. In humans, this process occurs over approximately 10 days (Robaire and Hermo 1994). Therefore, we studied the relationship between air quality and sperm parameters at 0–9, 10–14, and 70–90 days before the date of semen collection, corresponding to epididymal storage, the development of sperm motility, and spermatogenesis, respectively (Johnson et al. 1997). Data generated from animal studies support the hypothesis that exposures at different times during spermatogenesis manifest as abnormalities of ejaculated spermatozoa (Chapin 1997; Hess 1998).

Sperm donors were chosen as study subjects because *a*) they are rigorously selected healthy fertile men who donate many semen samples over an extended period of time; *b*) they must adhere to rigid guidelines regarding semen collection; and *c*) the semen samples were analyzed in a single laboratory.

## Materials and Methods

### Sperm donors and semen analysis.

Semen analysis data were accessed from the California Cryobank, which maintains a large sperm donor bank in Los Angeles. Donors were included if they collected repeated semen samples over at least a 12-month period from January 1996 through December 1998 and resided, to the best of our knowledge, in the same ZIP code area during that time period. The study protocol was reviewed and approved by the Institutional Review Board (IRB) of the University of Southern California Keck School of Medicine. The requirement for formal informed consent was waived by the IRB because the data analyzed were generated from previously collected samples that had been collected for routine laboratory testing and were anonymous to the research investigators.

The sperm donors studied were young men who were healthy, without genetic or significant medical diseases, and did not smoke, use drugs, or drink heavily. Each donor completed an extensive medical/social questionnaire to rule out any underlying personal or family history of disease and underwent a careful history and physical examination. General laboratory tests were evaluated in addition to screening semen analyses, all of which were normal in order to qualify as a sperm donor. Data provided for this study included age, date of birth, and race of donor; dates of semen collection; and ZIP codes of residence at the time of the first donation.

Semen samples were collected by masturbation into sterile designated containers at the sperm bank site. In accordance with the guidelines of the California Cryobank, donors were instructed to collect the semen samples after 2–3 days of abstinence. They were asked to report if they became ill or required medications and were instructed not to donate semen samples during those times. The importance of adhering to the abstinence instruction was emphasized at the time the donor was recruited and throughout his tenure as a donor.

The semen samples were analyzed within 1 hr of collection. Technicians were trained to analyze the semen samples using standardized protocols based on the World Health Organization (WHO) guidelines published at the time of the analyses (WHO 1993).

After liquefaction and measurement of semen volume, the semen analysis was performed using the Makler Chamber (Haifa, Israel) to determine sperm concentration and motility. Sperm morphology was not available. Quality control is used routinely in this laboratory, and technicians undergo proficiency testing on a regular basis.

### Air quality and temperature data.

Air quality data for the same time period were obtained from Sonoma Tech., Inc. (Petaluma, CA). Data were provided for 10 km × 10 km grid areas in the air basin of Southern California and included O_3_ (24-hr average, parts per billion), NO_2_ (0600–1800 hr average parts per billion), CO (24-hr average, parts per million), components of PM_10_ (micrograms per cubic meter), and 24-hr minimum, average, and maximum temperature. O_3_, NO_2_, and CO were measured daily and PM_10_ once every 6 days (Gauderman et al. 2000). Each donor was assigned a grid location according to their ZIP code of residence at the time of their first donation. Cumulative pollutant exposures over biologically relevant time frames, along with daily average, minimum, and maximum levels of temperature averaged over the same three time periods, were calculated for each donor by summing the appropriate measures for their grid location.

### Statistical methods.

Exploratory analyses were conducted to examine demographic characteristics of study participants, and univariate regression models were fitted to identify factors that would contribute to the multivariate analysis (SAS Institute Inc. 1997).

Semen analysis data were log_10_ transformed before analysis to ensure normality. We used a linear mixed-effects model to model linear relationships between transformed semen analysis data and air quality measurements. This approach accounts for repeated measures and variation in baseline among donors. The following linear mixed model was used to model log average concentration (log total motile) for the *j*th donation from subject *i: Y_ij_* = α+ γ*_i_* + β*Z_ij_* + δ*^T^X_ij_* + ε*_ij_*. The γ*_i_* terms represent individual differences in log average concentration (log total motile) and are assumed to be normally distributed with mean 0 and variance σ_γ_^2^· βdenotes the effects of an air pollutant *Z*, and δ denotes the effects covariates *X_ij_* weather, time trend, and other adjustment factors. The error terms ε*_ij_* are normally distributed and independent across subjects. Within subjects, an exponential-decay correlation structure was assumed; that is, observations close together in time were assumed to be more correlated than those farther apart. The mixed-effects methodology allows for unequal number of measurements from subjects. The exponential decay error structure allows for any residual serial correlation, above and beyond the exchangeable correlation structure that is induced by the subject specific random intercept in the mixed-effects model (Diggle et al. 1994). Analyses were performed using SAS PROC MIXED (SAS Institute Inc. 1997).

Temperature and seasonality effects were estimated after adjusting for a base model, which included donor’s date of birth and age at donation. Season was fitted using indicator (dummy) variables for spring (April–June), summer (July–September), and fall (October–December). Thus, parameter estimates should be interpreted as a change in mean outcome relative to winter.

Univariate effects of average air quality measures (averaged over each of the three time periods) were estimated after adjusting for the base model described above plus season indicators (as described above) and temperature values that were averaged over the biologically important time period under consideration for the air quality measures (e.g., a model that assesses the effect of an air quality measure 0–9 days before day of data collection adjusts for temperature values averaged for the same time period). To assess whether temperature levels on the day of data collection have an acute independent and/or confounding effect on reproductive outcomes, the models were also fitted by adjusting for minimum, average, and maximum levels of temperature on the day of data collection.

## Results

### Donors.

Forty-eight donors (of 50 potential donors) fulfilled the criteria for inclusion in the study and donated at least 10 times during the time period when air quality data were available. Two donors did not donate during the required period and hence were excluded from the analysis. The excluded subjects were not different with respect to their demographics.

Demographic and other descriptive statistics are included in Table 1. The donors were homogeneous with respect to their ethnicity (in fact, all donors were of non-Hispanic white ethnicity, except for one who was of Hispanic ethnicity). The distribution of age at first on-study donation ranged from 19 to 35 years of age. The average sperm concentration was 87.5 × 10^6^ sperm/mL, and the average total motile sperm count was 191.4 × 10^6^ motile sperm per sample.

### Air quality data.

**Figure 1:**
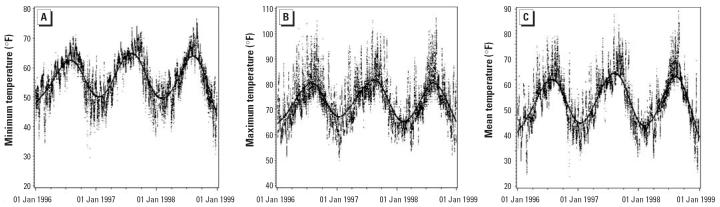
Daily ambient temperature measurements for Los Angeles from the grid locations. Solid lines are fitted cubic spline curves.

**Figure 2:**
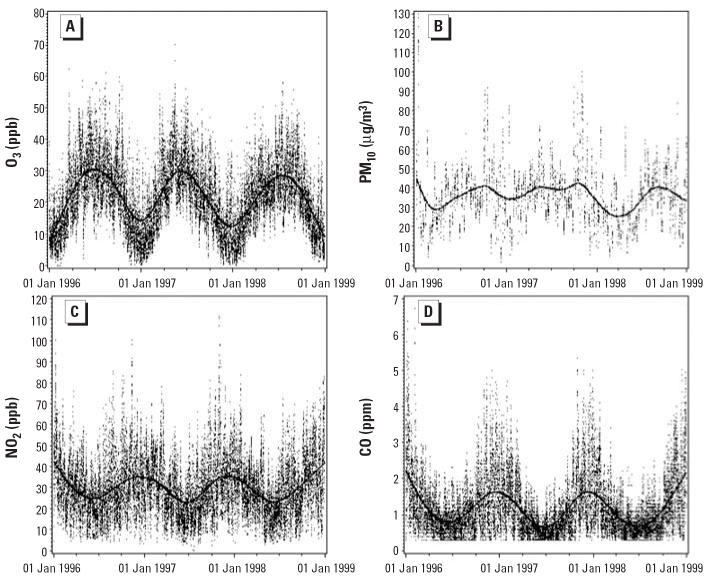
Daily air pollutant measures for Los Angeles from the grid locations. Solid lines are fitted cubic spline curves.

Table 2 and Figures 1 and 2 present 24-hr mean temperature and daily air quality, averaged over the grids where donors resided. Except for PM_10_, each measure showed clear seasonal variation.

Pollutant and temperature measurements were positively correlated for CO, NO_2_, and PM_10_ over the 0–9 day time period before ejaculation. CO and NO_2_ were negatively correlated with O_3_. PM_10_ was weakly positively correlated. O_3_ was positively correlated with daily minimum, mean, and maximum temperature (data not shown). The correlations between the average pollutants and temperature measurements followed a similar pattern for 10–14 and 70–90 days before ejaculation (data not shown).

To examine the effect of temperature on the day of ejaculation, models that included temperature levels on the day of ejaculation were fitted on the various pollutant and averaging-period combinations. These models showed that daily minimum, daily average, and daily maximum levels of temperature on the day of sperm donation were not independently associated with either average sperm concentration or total sperm motile (data not shown).

### Regression results.

**Figure 3:**
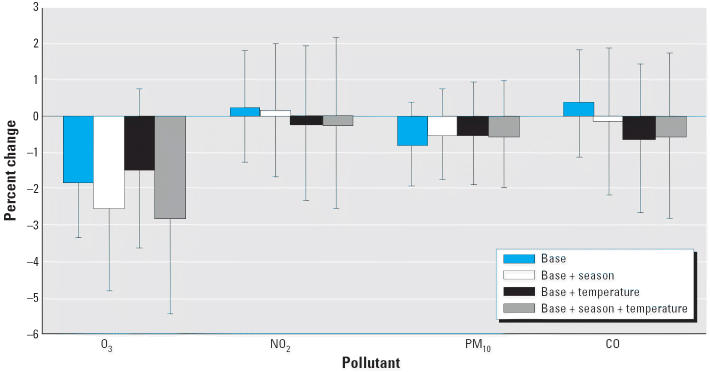
Percent change in sperm concentration for a 1 SD increase in air quality measure (lag 0–9 days). Error bars indicate 95% confidence intervals.

**Figure 4:**
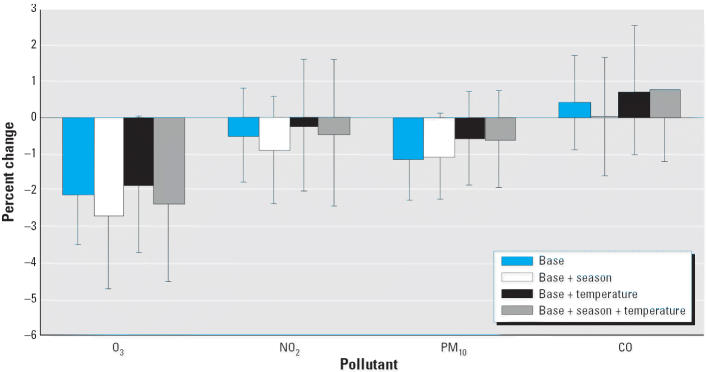
Percent change in sperm concentration for a 1 SD increase in air quality measure (lag 10–14 days). Error bars indicate 95% confidence intervals.

**Figure 5:**
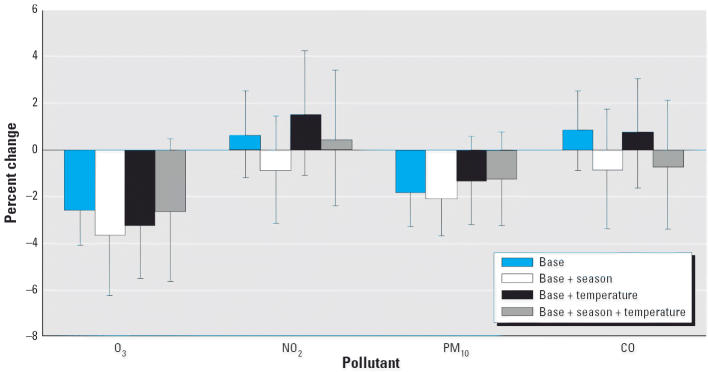
Percent change in sperm concentration for a 1 SD increase in air quality measure (lag 70–90 days). Error bars indicate 95% confidence intervals.

Univariate modeling of the potential confounders such as age, temperature, and season revealed that both average sperm count and total motile sperm count decreased significantly with age (data not shown). There was no significant univariate association observed for season and temperature, with the exception of season, which showed significant deficit in average sperm count in summer and fall (compared with winter; data not shown). Figures 3–5 summarize the associations between air quality measures and sperm quality parameters, after adjusting for several different sets of covariates (the base model of date of birth and age; this base model plus season; the base model plus temperature; the base model plus both season and temperature).

Several models gave evidence that an increase in O_3_ levels was associated with a decrease in sperm quality. A statistically significant association between O_3_ and average sperm count at all time periods was found when only adjusting for the base model. This association remained significant after adjustment for season and temperature [for 0–9 day lag, an estimated 2.80% decrease per interquartile range (IQR) of 14.3 ppb increase in O_3_, *p* = 0.04; for 10–14 day lag, an estimated 2.36% decrease per IQR of 14.3 ppb increase in O_3_, *p* = 0.04]. Although there was an estimated 2.61% decrease per IQR of 14.3 ppb for 70–90 day lagged O_3_ exposure, this did not reach statistical significance (*p* = 0.10). No relationship between O_3_ exposure and total motile sperm count was noted for any time period after adjusting for season and temperature.

No other pollutants were significantly associated with sperm quality measurements after correction for base model, temperature, and seasonality. Under a joint additive model for all four pollutants with adjustments for both temperature and season, the effects of O_3_ on average sperm concentration still persisted. For 0–9 days before semen collection, there was a 4.22% decrease per IQR of 14.3 ppb increase in O_3_ (*p* = 0.01). There were 2.92% and 3.90% decreases per IQR of 14.3 ppb increase in O_3_ 10–14 days and 70–90 days before day of ejaculation, respectively (*p* = 0.05 in both cases).

## Discussion

Our study was designed to examine the hypothesis that exposure to air pollutants may adversely affect semen quality and to ascertain when in the spermatogenesis cycle the toxicity may have occurred. We found that an inverse relationship exists between ambient O_3_ levels and sperm concentration at all biologic time periods studied. These results remain significant after adjustment for the potential confounders of age, ambient temperature, and seasonality. Our findings are substantiated by a study conducted in the Czech Republic by scientists from the U.S. Environmental Protection Agency (EPA). Young Czech men exposed to elevated air pollution were more likely to have abnormal sperm morphology and sperm chromatin structure than were those who lived in a city with less air pollution (Selevan et al. 2000; Sram et al. 1999). Although a decrease in sperm concentration was not found in the Czech study, this is probably due to differing experimental designs. The Czech study did not evaluate O_3_, restricted evaluation of the air pollution data to the 90-day period preceding sampling, and categorized the grouped pollutants as low, medium, and high. Only two semen specimens 6 months apart in men of unknown semen quality were studied. In our study design, we evaluate the relationship between specific air pollutants and sperm concentration and motility using repeated semen samples collected by men selected for the quality of their sperm over a 2-year period, with special emphasis on important developmental steps during the sperm production cycle and specific air pollutants. The toxic effects we report are specific to O_3_.

Potential confounders include temperature, seasonality, age, ethnicity, socioeconomic status, abstinence period, and method of semen analysis (Kandeel and Swerdloff 1999; Kidd et al. 2001; Levine 1994; Mahmoud et al. 1997; Paulson et al. 2001; Sauer et al. 1988). Our statistical model controlled for temperature, seasonality, age at donation, and date of birth. We cannot directly address the potential confounding effects of ethnicity or socioeconomic status because all but one of the donors in our study were educated non-Hispanic white men. However, differences in the prevalence of infertility across education levels or racial/ethnic categories in the United States have not been confirmed (Abma et al. 1997).

Our study controlled for semen analysis methodology, although technicians may have used minimally different semen analysis techniques, particularly in motility assessment. The technique for motility assessment outlined in the WHO guidelines at the time of this study (WHO 1993) is not a strictly quantifiable one, and it is possible that if a computer assisted sperm analysis system had been used to assess motility, we may have found significant differences in the total motile sperm counts due to O_3_ exposure. We controlled for abstinence period, but it is possible that donors may have had shorter or longer abstinence times than the ones they reported. However, we do not think that abstinence times differing from those requested affected our conclusions. Most studies evaluating between-subject variation in semen parameters report that semen volume and sperm concentration increase with increasing duration of abstinence, primarily after 5 days (Carlsen et al. 2004; De Jonge et al. 2004; Swan et al. 2003a). In our study, we evaluated repeated semen samples collected over time and thus looked at within-subject variation as well as the relationship of any changes in semen quality to air pollutant exposure. We previously reported, using an intrasubject study design, that semen volume and concentration were positively correlated with abstinence period only after 5 days of abstention (Sauer et al. 1988). Duration of abstinence and ejaculatory frequency have been reported to have little impact on high intraindividual variation in individual semen parameters (De Jonge et al. 2004). These data suggest that shorter abstinence time than reported does not account for our findings. Increasing abstinence time from 2.5 to 6 days has been estimated to increase sperm concentration by 50% (Carlsen et al. 2004). Therefore, an abstinence period > 3 days would not account for our finding of lower sperm concentrations on high O_3_ days.

We speculate that most donors spent most of their time in the ZIP code of residence, attending school or working in the local area. Nonetheless, some donors may have spent some time away from their assigned ZIP codes. We were not able to control for socioeconomic status; however, as indicated, most donors lived or worked near the university, which is located in a higher socioeconomic neighborhood not known for environmental toxic exposures.

Although some confounding may remain uncorrected for, we think that it is unlikely to explain the inverse relationship between O_3_ exposure and sperm concentration. Because air quality is related to geographic location, our data support the theory that chemical exposures may account for declining sperm densities in specific geographic regions (Fisch et al. 1996; Jorgensen et al. 2001; Swan et al. 2003a) and implicate O_3_ as a possible toxicant. Geographic differences in semen quality have been reported both in the United States and Europe. In an early study, sperm concentrations recorded in samples collected for a sperm bank in New York were reported to be higher than sperm bank samples in Minnesota, which were higher than those in California (Fisch et al. 1996). Regional differences in semen quality in Europe are also documented (Jorgensen et al. 2001). Pesticide exposure has been implicated as a possible etiology for the lower semen quality in fertile men in specific cities in the United States (Nelson and Bunge 1974; Swan et al. 2003a, 2003b). We suggest that O_3_ may also be implicated as a sperm toxicant.

O_3_ is the major oxidant of photochemical smog. It is a secondary pollutant, generated in the troposphere from the precursors NO_2_ and hydrocarbons in the presence of sunlight, which accounts for the observed negative correlation between O_3_ and NO_2_. O_3_ varies with temperature, cloudiness, and air circulation patterns (Mustafa 1990; U.S. EPA 2002). O_3_ is very reactive, with most of its toxic effects directed at the lung, with indirect toxicity on the cardiovascular system (Mann et al. 2002). O_3_ exposure produces reactive oxygen species (ROS) in the respiratory system (Gurgueira et al. 2002; Menzel 1984). Extrapulmonary toxicity suggests that O_3_ or O_3_ reaction products can cross the blood–gas barrier and be absorbed into the circulating bloodstream. These by-products may induce an inflammatory reaction or produce circulating toxic species (Gurgueira et al. 2002; National Toxicology Program 1994; Mustafa 1990).

How O_3_ may adversely affect semen quality remains to be elucidated. O_3_-induced oxidative stress is one possible mechanism. Oxidative stress is documented to disrupt testicular and sperm function (Agarwal et al. 2003; Diemer et al. 2003). Under physiologic conditions, spermatozoa exist in a balanced environment of ROS and antioxidants. ROS are needed for capacitation and the acrosome reaction, the biochemical/physiologic steps required for normal fertilization. However, excessive amounts of ROS produced by leukocytes and immature spermatozoa can damage mature spermatozoa and can damage the integrity of the DNA in the sperm nucleus (Agarwal et al. 2003; Aitken and Fisher 2004; Sikka et al. 1995).

Cigarette smoking can cause a modest decrease in sperm concentration, which is associated with ROS production in the respiratory system and increased seminal leukocyte infiltration into semen of infertile smokers and increased ROS in the semen (Saleh et al. 2002; Vine et al. 1994). As with smoking, exposure to O_3_ may either induce an inflammatory reaction in the male genital tract or induce the formation of circulating toxic species, both mechanisms that can lead to leukocytosis, ROS formation, spermatozoa phagocytosis, and a decline in sperm concentration. This ROS-induced DNA damage may accelerate the process of germ cell apoptosis (programmed cell death) and lead to a decline in sperm concentrations (Agarwal et al. 2003). Irradiation, chemotherapy, and toxin exposure have all been associated with apoptosis (Agarwal et al. 2003). A similar mechanism may be occurring with O_3_ exposure. Gaseous pollutants suppress spermatogenesis in exposed rats (Watanabe and Oonuki 1999). No studies have looked at the association between cigarette smoking, pollution, and semen quality. The reported finding of abnormal sperm morphology and sperm chromatin in semen samples collected by Czech men exposed to elevated air pollution is consistent with increased ROS in the semen samples of the men studied (Selevan et al. 2000). Although excess ROS may disrupt sperm motility, we did not find significant changes in total motile sperm counts with O_3_ exposure. This may be due to our relatively insensitive methodology for assessing motility, as discussed above. Some evidence of adverse effects of air pollution on sperm motility was reported in the Czech study (Selevan et al. 2000).

Studies in females have also suggested a relationship between air pollution and reproductive outcomes. Poor air quality may be associated with reduced pregnancy rates in *in vitro* fertilization centers (Boone et al. 1999), and exposure to increased levels of ambient air pollution is associated with preterm birth in Los Angeles, China, and the Czech Republic (CDC 2001; Ritz et al. 2000; Sram et al. 1999).

In summary, we noted an inverse relationship between O_3_ exposure and sperm concentration at all time points studied, suggesting that spermatozoa are susceptible to this toxic exposure throughout spermatogenesis. Overall, this study is well controlled for potential confounders, and the association between O_3_ and sperm quality is consistent across several models. No similar association between sperm density and exposure to the other air pollutants was found, further implicating O_3_ as a reproductive toxicant. We find these results very intriguing and worthy of further study.

## Figures and Tables

**Table 1:** Donor information (*n* = 48).

Donor/donation	Value
Sperm donations	
Total	5,134
Per donor (range)	20–207
Mean ± SD	135.4 ± 48.5
Median	145.0
Donor age at first donation	
Mean ± SD	25.3 ± 4.7
Median	24.0
Range	19–35
Average sperm concentration (10^6^/mL)	
Mean ± SD	87.5 ± 25.0
Median	83.0
Range	52.5–181.3
Total motile sperm count (× 10^6^)	
Mean ± SD	191.4 ± 49.2
Median	179.9
Range	130.5–370.3

**Table 2:** Daily average pollutant and temperature measurements.

Pollutant/temperature	Mean ± SD	Range	No.
O_3_ (ppb)	21.68 ± 9.43	1.69–47.51	1,096
NO_2_ (ppb)	30.11 ± 10.73	9.04–79.80	1,096
PM_10_ (μg/m^3^)	35.74 ± 13.83	6.84–101.88	183
CO (ppm)	1.18 ± 0.65	0.37–3.86	1,096
Minimum temperature (°F)	56.44 ± 7.37	35.86–74.18	1,096
Mean temperature (°F)	64.03 ± 7.12	45.50–83.37	1,096
Maximum temperature (°F)	73.58 ± 8.75	52.68–100.90	1,096
